# Validation of IMU-Based Insoles (LUBU) for the Estimation of Gait Spatio-Temporal Parameters

**DOI:** 10.3390/s26154792

**Published:** 2026-07-28

**Authors:** Chiara Orsanigo, Filippo Motta, Alessandro Giuseppe Milazzo, Manuela Galli

**Affiliations:** 1 Department of Electronics, Information and Bioengineering, Politecnico di Milano, Piazza Leonardo da Vinci 32, 20133 Milan, Italy; chiara.orsanigo@polimi.it (C.O.); filippo.motta@polimi.it (F.M.); alessandrogiuseppe.milazzo@mail.polimi.it (A.G.M.); 2 Department of Occupational and Environmental Medicine, Epidemiology and Hygiene, Italian National Institute for Insurance Against Accidents at Work (INAIL), Via Fontana Candida 1, Monte Porzio Catone, 00078 Rome, Italy

**Keywords:** validation, gait analysis, IMU-based insoles, LUBU insoles

## Abstract

**Highlights:**

**What are the main findings?**

- LUBU insoles showed low errors in the estimation of gait events and spatio-temporal parameters.
- Agreement with the optoelectronic reference system was good to excellent for the parameters investigated.

**What are the implications of the main findings?**

- LUBU insoles may represent a practical wearable tool for spatio-temporal gait analysis.
- The system may help extend gait analysis from controlled laboratories to more ecological walking conditions.

**Abstract:**

Gait analysis is a well-known tool used to evaluate human locomotion. This analysis is usually performed in movement analysis laboratories equipped with optoelectronic cameras. However, many wearable devices based on the use of Inertial Measurement Units (IMUs) have been developed to overcome the limitations of the gold standard technology. LUBU Technologies developed insoles equipped with IMUs and algorithms for the extraction of gait parameters such as gait events, temporal parameters and stride length. The aim of the present study was the validation of these parameters against an optoelectronic reference system. Twenty people were involved in performing a series of indoor walking trials to assess the accuracy of the insole measurements and an additional group of ten people were asked to walk outdoors to preliminarily assess total distance estimation under ecological walking conditions. The results showed low errors and good agreement for all the parameters computed by the insoles. Moreover, the outdoor assessment offered preliminary insights into performance in a more ecological setting. Overall, the findings suggest that LUBU insoles may represent a valid wearable tool for estimating gait spatio-temporal parameters in healthy young adults. However, further investigations are required in older adults or populations with pathological gait patterns.

## 1. Introduction

Gait analysis is a common tool used to quantify human locomotion outcomes such as spatio-temporal and kinematic parameters [1]. Optoelectronic cameras are considered the technological gold standard in gait analysis, providing reliable and accurate measurement of movement [2]. These systems find many applications in the medical field, ranging from diagnostic support [3] to the supervision of rehabilitation programs [4]. However, the use of this technology presents some disadvantages. Firstly, optoelectronic cameras are used in specific laboratories, limiting the possibility of movement evaluations in more realistic settings [5,6]. Moreover, the complexity of the system requires technical expertise both for the positioning of reflective markers and for the interpretation of outputs.

Great effort has been put into the development of new tools for gait analysis to overcome the disadvantages of the gold standard. The most widespread approach is the use of Inertial Measurement Units (IMUs) combined with software and algorithms to measure the movement parameters of the segment on which they are positioned [7]. These systems are developed to be compact and easy to use, making them a good solution for monitoring everyday activities [8]. Among the multiple solutions available in terms of IMU placement, the idea of using sensorized insoles that can be placed directly inside everyone’s shoes is quite widespread and many studies highlight the reliability of their measurements [9,10,11,12,13,14]. Although several IMU-based insoles have been proposed and validated for gait analysis, the performance of these systems is strictly dependent on the device and its algorithm. The accuracy of the extracted gait parameters can be affected by sensor placement, sampling frequency, signal processing and event detection strategy [15]. Thus, validation results obtained for one commercial insole cannot be directly generalized to other systems [9].

LUBU insoles represent a commercial wearable device designed to provide relevant information such as gait spatio-temporal parameters. To our knowledge, gait spatio-temporal parameters obtained from these insoles have never been validated against a reference system. Thus, the aim of this study was to assess the accuracy and the absolute agreement of gait parameters from LUBU insoles compared to parameters coming from an optoelectronic system.

## 2. Materials and Methods

LUBU insoles (LUBU Technologies Inc., Los Angeles, CA, USA) are provided with an IMU with a sampling frequency of 200 Hz (LSM6DSO32, STMicroelectronics, Geneva, Switzerland) placed under each insole in the mid-foot area. To obtain gait spatio-temporal parameters from the IMU signals, a Butterworth low-pass filter with a cut-off frequency of 10 Hz was applied. The signal of the gyroscope around the mediolateral axis was used to identify heel strike (HS) and toe-off (TO) instants. Using HS and TO event identification, the main temporal parameters of gait were computed. Thus, beyond HS and TO instants, LUBU insoles give as output swing time (SWT), stance time (STT) and cycle duration (CD) defined as the time between two consecutive TOs of the same foot. Furthermore, quaternions were computed from the inertial sensor data, following the method presented by Rampp et al. [16], to compute the stride length (SL) defined as the distance covered during a gait cycle.

To validate the information coming from the insoles, data collection was performed both indoors and outdoors. Twenty people (13 males, 7 females; age: 24.75 ± 1.33) were involved in the indoor acquisitions while ten (7 males, 3 females; age: 25.5 ± 3.37) were asked to take part in the outdoor data collection. For both conditions, a specific protocol was followed. All participants provided written informed consent to participate in the study. The study was carried out in accordance with the Declaration of Helsinki and the protocol was approved by the Ethics Committee of Politecnico di Milano (ref number: 28/2026) on 23 April 2026.

### 2.1. Indoor Data Collection

**Figure 1:**
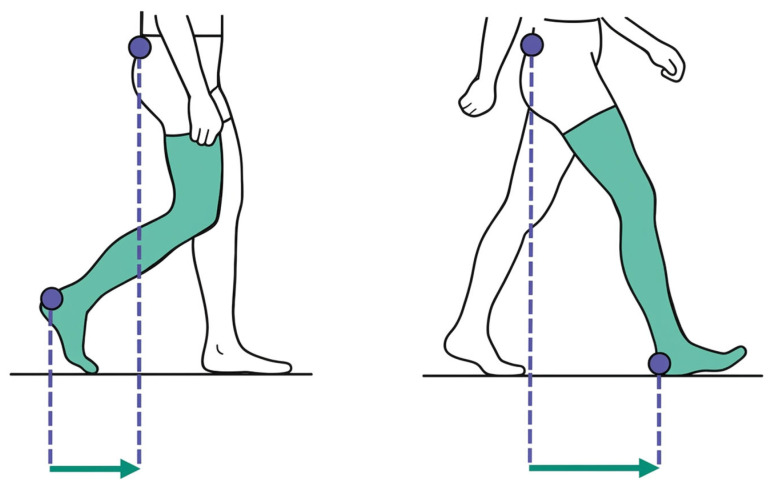
Gait temporal events identification from optoelectronic data. Heel strike on the left and toe-off on the right.

Indoor data were acquired in a movement analysis laboratory equipped with an eight-camera optoelectronic system (SMART DX400, BTS Bioengineering S.p.A., Garbagnate Milanese, Italy), with a sampling frequency of 100 Hz, that was used as the reference system. A pair of LUBU insoles of the correct size was put inside the participants’ shoes and three reflective markers were applied on them, specifically one for each heel and one on the pelvis, to detect gait parameters. The marker placement was sufficient for gait event identification. Heel strikes and toe-offs were identified using the distance between the markers on the heels and the marker on the pelvis. More precisely, TOs were identified as the moments at which the foot was behind the pelvis and the distance from the pelvis reached its maximum, while HSs were considered as the instants at which the foot was ahead of the pelvis and the distance from the pelvis reached its maximum [17]. The kinematic approach followed, visible in Figure 1, has been previously validated for gait event detection.

Once the participants were ready, they were asked to perform a single jump, to ensure the possibility of synchronization of signals, followed by 16 walking trials of eight steps each. Gait spatio-temporal information coming from the insoles was compared with the same data obtained from the optoelectronic system.

### 2.2. Outdoor Data Collection

To evaluate the insoles’ performance in a more realistic setting, people were asked to perform a 1275 m walk following a fixed path at a self-selected speed while wearing a correct-sized pair of sensorized insoles. The total distance walked, computed by the insoles as the sum of all the stride lengths performed during the walk, was then compared with the distance given by a GPS to assess the cumulative distance error under outdoor conditions. Since GPS provided only a reference for the total traveled distance, stride-by-stride validation was not possible in the outdoor protocol. Thus, for each participant, a regression line was fitted to the stride length values estimated during outdoor walking as a function of the stride index to evaluate possible progressive changes in the estimated stride length over the acquisitions.

### 2.3. Statistical Analysis

Statistical analysis was performed using MATLAB R2025a (The MathWorks, Inc., Natick, MA, USA). The distribution of each variable was assessed for normality using the Lilliefors test. To evaluate the accuracy of the proposed method relative to the gold standard, the median absolute error (MedAE), interquartile range (IQR), and root mean square error (RMSE) were computed for all variables of interest; for spatial outcomes, the median percentage error was also calculated. Agreement between the two systems was further investigated using Bland–Altman analysis by computing the mean bias and the 95% limits of agreement (LoA) with their 95% confidence intervals (CIs). Moreover, the presence of proportional bias was assessed using the slope of the regression line between the differences in and the averages of the IMU and reference system measurements. In addition, the intraclass correlation coefficient (ICC) was calculated to assess agreement between the measurements obtained from the two systems. The ICC estimates and their 95% confidence intervals were calculated on subject-level mean values using a single-measurement, absolute-agreement, two-way random effects model [ICC(2,1)]. ICC values lower than 0.5, between 0.5 and 0.75, between 0.75 and 0.9, and greater than 0.9 were interpreted as poor, moderate, good, and excellent, respectively [18]. Moreover, Spearman’s correlation coefficient (ρ) was computed for the spatial and temporal parameters to assess the monotonic association between the estimated and reference measurements. The correlation was considered negligible if lower than 0.10, weak between 0.10 and 0.39, moderate between 0.40 and 0.69, strong between 0.70 and 0.89 and very strong if higher than 0.90 [19]. To further assess the agreement between LUBU insoles and the reference system, Lin’s concordance correlation coefficient (CCC) was computed for the temporal gait parameters and stride length [20]. CCC values were interpreted according to McBride criteria reported by Akoglu with values below 0.90 considered poor, values between 0.90 and 0.95 moderate, values between 0.95 and 0.99 substantial, and values above 0.99 almost perfect [21]. CCC values were interpreted together with bias and Bland–Altman limits of agreement to evaluate the practical acceptability of the observed differences.

## 3. Results

The total number of steps recorded during indoor data collection was 2560 but the first and the last two steps of each trial were discarded so the total number of steps considered to assess the accuracy of the LUBU insoles was 1600.

### 3.1. Accuracy of HS and to Detection

The results obtained for heel strike and toe-off timings (Table 1) showed low errors for both events, with a MedAE of 0.018 s (IQR: 0.008–0.030 s) for HS and 0.015 s (IQR: 0.010–0.025 s) for TO. Moreover, the RMSE resulted in 0.030 s for HS and 0.023 s for TO.

**Figure 2:**
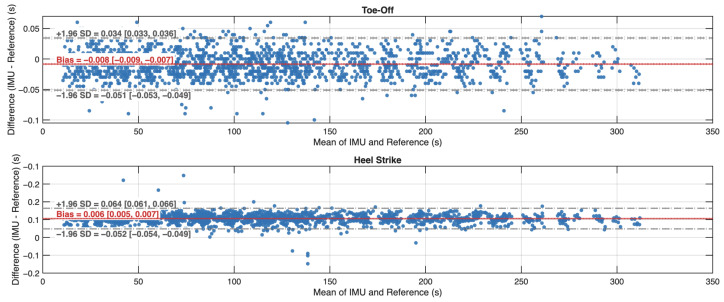
Bland–Altman plots showing the agreement between the sensorized insole-based estimates and the optoelectronic reference system for toe-off and heel strike instants. The red solid line indicates the mean bias, the gray dash-dotted lines indicate the upper and lower 95% limits of agreement (bias ± 1.96 SD). Values in brackets represent the corresponding 95% confidence intervals.

Biases and limits of agreement were evaluated through Bland–Altman analysis (Figure 2). For both events, biases were small (HS = 0.006 s, TO = −0.008 s) and limits of agreement were narrow (HS = [−0.052 s, 0.064 s], TO = [−0.051 s, 0.034 s]), indicating good consistency between the two systems. Linear regression of the differences against the averages revealed the absence of proportional bias for TO instants while a statistically significant proportional bias was observed for HS (slope = −3.35 × 10^−5^, *p* = 0.0012). However, the explained variance was low (R^2^ = 0.0067); thus, the proportional bias is likely of limited practical relevance.

### 3.2. Performance on Temporal Gait Parameters

The temporal gait parameters were then evaluated, including gait cycle duration, stance time, and swing time (Table 2).

**Figure 3:**
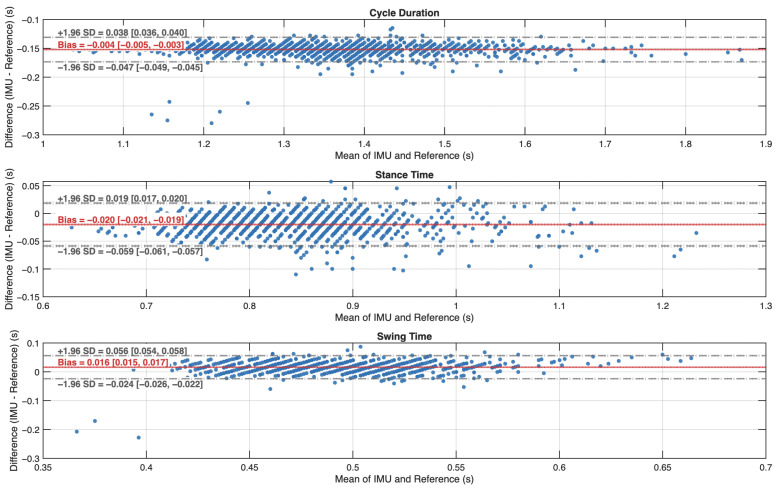
Bland–Altman plots showing the agreement between the sensorized insole-based estimates and the optoelectronic reference system for cycle duration, stance time and swing time. The red solid line indicates the mean bias, the gray dash-dotted lines indicate the upper and lower 95% limits of agreement (bias ± 1.96 SD). Values in brackets represent the corresponding 95% confidence intervals.

As for HS and TO events, temporal parameters were assessed using the MedAE and its interquartile range (CD = 0.010 s [0.005 s–0.015 s], SWT = 0.018 s [0.010 s–0.028 s], STT = 0.020 s [0.010 s–0.031 s]) and RMSE (CD = 0.022 s, SWT = 0.026 s, STT = 0.028 s). Bland–Altman analysis (Figure 3) was performed by computing biases and 95% limits of agreement for all temporal parameters (CD = −0.004 s [−0.047 s, 0.038 s], SWT = 0.016 s [−0.024 s, 0.056 s], STT = −0.020 s [−0.059 s, 0.019 s]), showing small biases and narrow limits of agreement. The proportional bias resulted in being statistically significant only for the SWT (slope = 0.081, *p* < 0.001); however, the explained variance was low (R^2^ = 0.023). Finally, correlation and absolute agreement with the reference system were assessed. Spearman’s correlation coefficients revealed very strong monotonic associations for the cycle duration (ρ = 0.98, *p* < 0.0001) and for the stance time (ρ = 0.97, *p* < 0.0001) while for the swing time, the correlation was strong (ρ = 0.88, *p* < 0.0001). Similarly, the ICC values indicated excellent agreement for the cycle duration (ICC = 0.99, *p* < 0.0001) and stance time (ICC = 0.96, *p* < 0.0001) and good agreement for the swing time (ICC = 0.85, *p* < 0.0001). The CCC values showed substantial concordance for the cycle duration (CCC = 0.984), moderate concordance for the stance time (CCC = 0.948), and poor concordance for the swing time (CCC = 0.793). This lower CCC for the swing time suggests that, despite the low absolute error, the agreement for this parameter was weaker than for the cycle duration and stance time.

### 3.3. Performance on Spatial Gait Parameters

For the spatial domain, stride length was evaluated against the reference system (Table 3).

**Figure 4:**
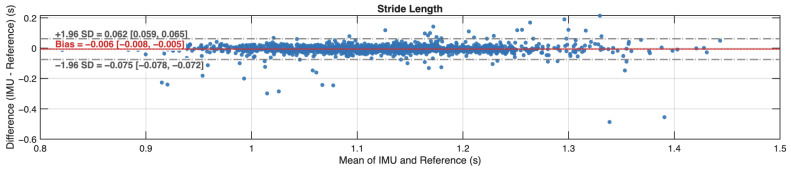
Bland–Altman plot showing the agreement between the sensorized insole-based estimates and the optoelectronic reference system for stride length. The red solid line indicates the mean bias, the gray dash-dotted lines indicate the upper and lower 95% limits of agreement (bias ± 1.96 SD). Values in brackets represent the corresponding 95% confidence intervals.

The proposed method achieved a MedAE of 0.012 m (IQR: 0.006 m–0.022 m), an RMSE of 0.036 m, and a median percentage error of 1.10% (IQR: 0.54–1.99%). Spearman’s correlation coefficient revealed a very strong monotonic association with the reference measurements (ρ = 0.96, *p* < 0.0001), while the ICC indicated excellent agreement (ICC = 0.99, *p* < 0.0001). CCC indicated moderate concordance (CCC = 0.926). Bland–Altman analysis showed a mean bias of −0.006 m, with limits of agreement ranging from −0.075 m to 0.062 m, as shown in Figure 4. No evidence of proportional bias was found for stride length.

**Figure 5:**
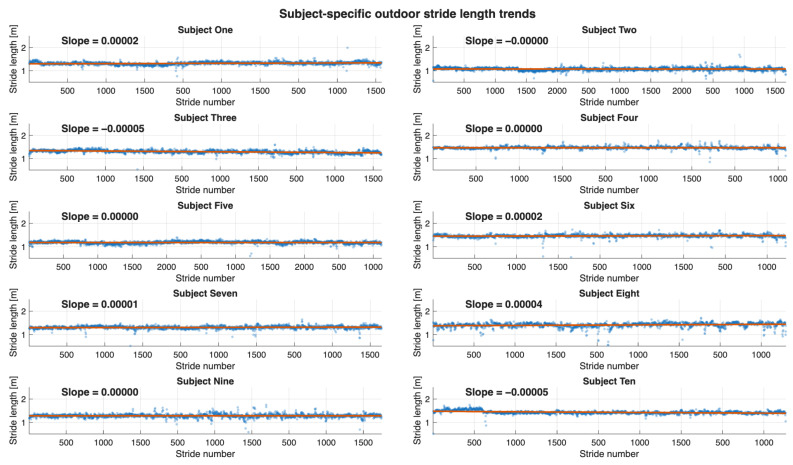
Linear regression analysis of stride length values for each participant during outdoor walking. Blue markers represent the stride length values while the red lines represent the fitted linear regression. For each participant, the angular coefficient of the regression line is reported in the corresponding graph.

To further assess the performance of the method, the total distance walked was evaluated during outdoor trials. The proposed system achieved a MedAE of 58.95 m (IQR: 54.47 m–60.84 m), an RMSE of 63.04 m, and a median percentage error of 4.62% (IQR: 4.27–5.56%). The regression analysis of the estimated stride length values of each participant is reported in Figure 5. The regression slopes were small, suggesting no evident progressive change in the estimated stride length over the tested path.

## 4. Discussion

The main objective of the study was to assess the accuracy and agreement of gait spatio-temporal parameters from LUBU insoles compared with gait parameters obtained from an optoelectronic system. Data were collected on 20 participants during a single session of overground walking. Moreover, 10 participants were asked to perform an outdoor walk along a fixed path at a self-selected speed to assess the accuracy of the spatial parameter in ecological conditions. Overall, the system showed good performance in indoor conditions for gait event detection, temporal gait parameters, and stride length estimation. This pattern is consistent with previous validation studies on instrumented insoles, where temporal gait parameters generally showed higher accuracy than spatial measures [9,10,11]. In outdoor conditions, total distance estimation was less accurate, but it still provided useful information in a more ecological walking context.

The practical acceptability of the observed errors should be interpreted in relation to the intended use of the system. For gait event detection, the limits of agreement were within approximately ±50–60 ms, while the MedAE values were below 20 ms for both HS and TO. These errors are small relative to the duration of the gait cycle and support the use of the system for estimating spatio-temporal gait parameters in healthy young adults under controlled walking conditions. For the temporal parameters, the MedAE values were between 10 and 20 ms, with RMSE values below 30 ms, suggesting adequate accuracy for practical gait assessment and monitoring of general spatio-temporal patterns. For stride length, the median absolute error was 0.012 m and the median percentage error was 1.10%, indicating low average error in the indoor protocol. However, the Bland–Altman limits of agreement for stride length ranged from −0.075 m to 0.062 m, corresponding to −6.6% and 5.5% of the typical stride length measured by the optoelectronic system, respectively. This indicates that, despite the small average bias, larger differences may occur at the single-stride level. Therefore, these limits should be considered when interpreting individual strides.

The correct timing identification of heel strike and toe-off instants is crucial because temporal parameters are strictly dependent on them. However, results about the correct identification of TO and HS are rarely reported in insole validation studies, which more frequently report the derived temporal parameters rather than the direct timing error of gait events themselves, even though HS and TO remain the basis for the computation of the main temporal outcomes. The method used by LUBU insoles, based on the gyroscope’s signal around the mediolateral axis, showed lower errors, smaller bias and similar limits of agreement compared with the results obtained by Huang et al. [10]. Thus, the choice of the gyroscope signal for event identification appears to be appropriate and robust.

The results achieved for the temporal parameters (cycle duration, stance time and swing time) are a direct consequence of the good results obtained in the HS and TO event identification. In fact, for all temporal parameters, low errors, small biases and narrow limits of agreement were found. In this context, the results obtained with LUBU insoles appear fully in line with the literature [10,22] and, for cycle duration, even slightly better than some previously reported ICC values [11]. Cycle duration showed the best results among the temporal parameters. This can be explained by the fact that the cycle duration was computed as the difference between two consecutive TOs of the same foot, with TO showing a slightly lower MedAE and RMSE than HS. Heel strikes, on the other hand, are involved in the computation of the swing time and stance time. Moreover, the strong correlation and excellent ICC value indicate a high agreement between the insoles and the reference system, at least for the walking trials performed in the indoor protocol.

The interpretation of agreement metrics deserves specific consideration. Spearman’s correlation coefficient was not interpreted as evidence of agreement. Agreement was instead evaluated using Bland–Altman analysis, absolute-agreement ICC, and CCC. This distinction is relevant because two methods may be highly correlated while still presenting systematic or proportional differences. In the present study, the ICC values were good to excellent for the investigated temporal and spatial parameters, while CCC provided a more conservative estimate of concordance, particularly for the swing time and stride length. This suggests that LUBU insoles reliably followed the reference measurements, but that the level of concordance varied depending on the parameter considered.

The low errors, small biases, and narrow limits of agreement found for stride length support the accuracy of the method used for the computation of spatial parameters from LUBU insoles [16]. Moreover, excellent ICC and moderate CCC ensure a good agreement of the insoles with the reference system at least for the walk performed in the indoor protocol. Considering previous validation studies on instrumented insoles, stride length estimation has shown variable performance, with some studies reporting lower agreement than temporal gait parameters [9,10,22], while others have reported excellent validity [11]. Table 4 summarizes the main methodological characteristics and validation outcomes of representative instrumented insole systems.

Finally, the median percentage error found for the total distance (4.62%) performed in the outdoor protocol, obtained as the sum of all the stride lengths computed during the task execution, was higher than the median percentage error computed on each stride (1.10%) performed in the indoor data collection. However, the regression analysis of the stride length values estimated during outdoor walking did not suggest an evident progressive change over time (Figure 5). Thus, the higher percentage error obtained for the outdoor data collection may be related to the environment in which the walking was performed. In fact, walking outdoors involves the presence of other people, the need to perform changes in direction to follow the fixed path and the presence of an uncontrolled surface. Moreover, since stride-by-stride validation was not possible in the outdoor protocol, the presence of systematic bias in stride length estimation cannot be excluded. All these elements can be considered as sources of possible errors in the stride length computation affecting the total distance estimation.

The proposed study presents some limitations. First, the population included in the study was relatively homogeneous, as it consisted only of healthy young adults, reducing the generalizability of the findings. Gait patterns in elderly and pathological individuals may differ from those observed in young healthy adults, possibly affecting both gait event detection and stride length estimation. In addition, for the indoor protocol, only the central strides of each trial were considered, possibly reducing the variability in the gait patterns included in the accuracy assessment. Second, no a priori sample size calculation or power analysis was performed. Future validation studies should include formal sample size planning based on predefined acceptable limits of agreement. Third, regarding the outdoor protocol, GPS was used as an external reference for total distance, but GPS measurements are affected by positioning uncertainty and cannot be considered equivalent to a laboratory reference system. Moreover, all participants performed a path of the same length; thus, it was not possible to investigate how the error in total distance estimation changes depending on the distance covered. Future studies should consider more heterogeneous populations, possibly including older adults or individuals with a pathological gait. Moreover, outdoor protocols including paths of longer distances and different surfaces would allow a more complete investigation of the behavior of the error in total distance estimation.

## 5. Conclusions

Gait parameters coming from LUBU insoles were compared with data obtained from an optoelectronic reference system in healthy young adults. The results obtained in a controlled environment showed low errors and high agreement of the insole-based measurements for gait event detection, temporal and spatial parameters, supporting the validity of the device under investigation in controlled walking tasks. Moreover, the outdoor assessment suggested no evident progressive change in stride length estimation during a fixed distance walk, but the protocol does not allow definitive conclusions on long-term drift-free performance. Therefore, LUBU insoles may represent a promising wearable tool for spatio-temporal gait assessment in healthy young adults. However, these findings should not be generalized to elderly individuals or patients with pathological gait patterns without further dedicated validation studies.

## Figures and Tables

**Table 1:** Accuracy and agreement of gait event detection performed by LUBU insoles compared with the optoelectronic reference system during the indoor walking protocol. MedAE: median absolute error; IQR: interquartile range; RMSE: root mean square error; LoA: limits of agreement.

	Errors			Bland–Altman		
	MedAE [95% CI]	IQR	RMSE [95% CI]	Bias [95% CI]	Lower LoA [95% CI]	Upper LoA [95% CI]
**TO [s]**	0.015 [0.015–0.020]	0.010–0.025	0.023 [0.020–0.026]	−0.008 [−0.009– −0.007]	−0.051 [−0.053– −0.049]	0.034 [0.032– 0.036]
**HS [s]**	0.018 [0.013–0.023]	0.008–0.030	0.030 [0.025–0.035]	0.006 [0.005– 0.007]	−0.052 [−0.054– −0.049]	0.064 [0.061– 0.066]

**Table 2:** Accuracy and agreement of temporal gait parameters measured by LUBU insoles compared with the optoelectronic reference system during the indoor walking protocol. MedAE: median absolute error; IQR: interquartile range; RMSE: root mean square error; LoA: limits of agreement; ICC: intraclass correlation coefficient; CCC: Lin’s concordance correlation coefficient. * indicates *p*-value < 0.001.

		Cycle Duration [s]	Swing Time [s]	Stance Time [s]
**Errors**	*MedAE [95% CI]*	0.010 [0.010–0.010]	0.018 [0.015–0.020]	0.020 [0.017–0.025]
	*IQR*	0.005–0.015	0.010–0.028	0.010–0.031
	*RMSE [95% CI]*	0.022 [0.016–0.029]	0.026 [0.022–0.030]	0.028 [0.024–0.032]
**Bland**–**Altman**	*Bias [95% CI]*	−0.004 [−0.005– −0.003]	0.016 [0.015– 0.017]	−0.020 [−0.021– −0.019]
	*Lower LoA* *[95% CI]*	−0.047 [−0.049– −0.045]	−0.024 [−0.026– −0.022]	−0.059 [−0.061– −0.057]
	*Upper LoA* *[95% CI]*	0.038 [0.036– 0.040]	0.056 [0.054– 0.058]	0.019 [0.017– 0.020]
**Spearman’s coefficient**	*ρ*	0.98 *	0.88 *	0.97 *
**ICC** **(2,1)**	*ICC* *[95% CI]*	0.998 * [0.994–0.999]	0.854 * [0.702–0.912]	0.961 * [0.908–0.977]
**Lin’s concordance correlation**	*CCC*	0.984 [0.959–0.993]	0.793 [0.670–0.863]	0.948 [0.902–0.968]

**Table 3:** Accuracy and agreement of stride length measured by LUBU insoles compared with the optoelectronic reference system during the indoor walking protocol. MedAE: median absolute error; IQR: interquartile range; RMSE: root mean square error; LoA: limits of agreement; ICC: intraclass correlation coefficient; CCC: Lin’s concordance correlation coefficient. * indicates *p*-value < 0.001.

		Stride Length [m]
**Errors**	*MedAE [95% CI]*	0.012 [0.010–0.015]
	*IQR*	0.006–0.022
	*RMSE [95% CI]*	0.036 [0.023–0.047]
**Bland**–**Altman**	*Bias [95% CI]*	−0.006 [−0.008–−0.005]
	*Lower LoA* *[95% CI]*	−0.075 [−0.078–−0.072]
	*Upper LoA* *[95% CI]*	0.062 [0.059–0.065]
**Spearman’s coefficient**	*ρ*	0.96 *
**ICC** **(2,1)**	*ICC* *[95% CI]*	0.989 [0.971–0.994]
**Lin’s concordance correlation**	*CCC*	0.926 [0.862–0.966]

**Table 4:** Comparison between LUBU insoles and representative published instrumented insole systems for gait spatio-temporal parameter validation. NR: not reported; HS: heel strike; TO: toe-off; CD: cycle duration; SWT: swing time; STT: stance time; ICC: intraclass correlation coefficient; CCC: Lin’s concordance correlation coefficient; RMSE: root mean square error; MAE: mean absolute error; MedAE: median absolute error, LoA: limits of agreement.

		LUBU	DSPro (Riglet 2023) [9]	FeetMe (Huang 2025) [10]	PODOSmart (Ziagkas 2021) [11]	Insole3 (Ganguly 2023) [22]
**Sample size**		20 healthy young adults	30 healthy adults	37 healthy adults	11 healthy male adults	12 healthy adults
**Sensor configuration**		IMU	IMU	IMU + pressure insole	IMU	IMU + 16 pressure sensors
**Environment**		Overground walking	Overground + treadmill, 3 speeds	Overground walking	Overground walking	Overground walking, 2 speeds
**HS**	*MedAE or MAE*	0.018 s	NR	0.025 s	NR	NR
	*RMSE*	0.030 s	NR	NR	NR	NR
	*Bias [LoA]*	0.006 s [−0.052–0.064 s]	NR	NR	NR	NR
**TO**	*MedAE or MAE*	0.015 s	NR	0.025 s	NR	NR
	*RMSE*	0.023 s	NR	NR	NR	NR
	*Bias [LoA]*	−0.008 s [−0.051–0.034 s]	NR	NR	NR	NR
**Temporal parameters**	*MedAE or MAE*	CD = 0.010 s SWT = 0.018 s STT = 0.020 s	NR	CD = 0.012 s SWT = 0.019 s STT = 0.021 s	NR	NR
	*RMSE*	CD = 0.022 s SWT = 0.026 s STT = 0.028 s	NR	NR	NR	CD = 0.016–0.018 SWT = 0.017–0.029 s STT = 0.016–0.033
	*ICC*	CD = 0.998 SWT = 0.854 STT = 0.961	CD > 0.96 SWT > 0.93 STT > 0.98	CD = 1 SWT = 1 STT = 1	CD = 0.97 SWT = 0.57 STT = 0.57	NR
	*CCC*	CD = 0.984 SWT = 0.793 STT = 0.948	NR	NR	NR	NR
**Stride length**	*MedAE or MAE*	0.012 m	NR	0.06 m	NR	NR
	*RMSE*	0.036 m	NR	NR	NR	0.108–0.205 m
	*ICC*	0.989	>0.96	0.73	0.94	NR
	*CCC*	0.926	NR	NR	NR	NR

## Data Availability

The data presented in this study are available on request from the corresponding author.

## Abbreviations

The following abbreviations are used in this manuscript:

IMU	Inertial Measurement Units
TO	Toe-off
HS	Heel strike
SWT	Swing time
STT	Stance time
CD	Cycle duration
SL	Stride length
MAE	Mean absolute error
MedAE	Median absolute error
IQR	Interquartile range
RMSE	Root mean square error
LoA	Limits of agreement
CI	Confidence interval
ICC	Intraclass correlation coefficient
CCC	Lin’s concordance correlation coefficient
